# E-cadherin expression pattern during zebrafish embryonic epidermis development

**DOI:** 10.12688/f1000research.15932.3

**Published:** 2019-02-18

**Authors:** María Florencia Sampedro, María Fernanda Izaguirre, Valeria Sigot

**Affiliations:** 1 Laboratorio de Microscopía Aplicada a Estudios Moleculares y Celulares (LAMAE), Facultad de Ingeniería, Universidad Nacional de Entre Ríos, Oro Verde, 3100, Argentina; 2 Instituto de Investigación y Desarrollo en Bioingeniería y Bioinformática (IBB-CONICET- Universidad Nacional de Entre Ríos), Oro Verde, 3100, Argentina

**Keywords:** Key words: E-cadherin; adherens junctions; zebrafish epidermis; enveloping layer (EVL), epidermis basal layer (EBL), deconvolution, 3D segmentation

## Abstract

**Background**: E-cadherin is the major adhesion receptor in epithelial adherens junctions (AJs). On established epidermis, E-cadherin performs fine-tuned cell-cell contact remodeling to maintain tissue integrity, which is characterized by modulation of cell shape, size and packing density. In zebrafish, the organization and distribution of E-cadherin in AJs during embryonic epidermis development remain scarcely described.

**Methods:** Combining classical immunofluorescence, deconvolution microscopy and 3D-segmentation of AJs in epithelial cells, a quantitative approach was implemented to assess the spatial and temporal distribution of E-cadherin across zebrafish epidermis between 24 and 72 hpf.

**Results: **increasing levels of E-cadh protein parallel higher cell density and the appearance of hexagonal cells in the enveloping layer (EVL) as well as the establishments of new cell-cell contacts in the epidermal basal layer (EBL), being significantly between 31 and 48 hpf **.**

**Conclusions:** Increasing levels of E-cadherin in AJs correlates with extensive changes in cell morphology towards hexagonal packing during the epidermis morphogenesis.

## Introduction

The skin is the largest organ of the body in direct contact with the environment. It has a complex structure, being constituted by many different tissues. Skin performs functions that are vital in maintaining body homeostasis, such as the control of body temperature and protection from physical damage and bacterial invasion. Sensory axons innervate the skin at early developmental stages enabling the embryo to sense mechanical, thermal, and chemical stimuli ^1^.

In particular, the topology –connectivity, continuity and neighborhood–, and individual cell phenotypes of epidermis define the strength and permeability of the protective barrier between the organism and its environment. Although epidermis structure varies between aquatic and terrestrial organisms, its stratification is a common mechanism during development ^2^.

### Epidermis development in zebrafish (Danio rerio)

In zebrafish, an immature epidermis establishes soon after the end of gastrulation, constituted by the surface layer, enveloping layer (EVL), and the inner epidermal basal layer (EBL) ^3, 4^. EVL arises at mid-blastula stage (2.5 hours post fertilization; hpf) covering the whole embryo ^5^. During epiboly EVL maintains tight joins to the yolk syncytial layer (YSL), and becomes the migration substrate for the underlying deep cells spreading during gastrulation ^6^. At the tail bud stage (8–10 hpf), the epiblast forms the outer germ layer, the ectoderm, characterized as a pseudo-epithelial germ layer. From the non-neural ectoderm arises the EBL, which covers the whole embryonic surface underneath the EVL to form a two-layered epithelium by 10 hpf. At 24 hpf, the epidermis is a distinctive bilayer in which the basal layer actively produces collagen to form the basal membrane and the primary dermal stroma ^7, 8^. It is after three weeks that the epidermis becomes further stratified and develops into the adult teleost four-layered epidermal structure: cuticle, surface, intermediate and basal stratums ^9^. In adult zebrafish, the EVL cells are replaced by those derived from basal keratinocytes ^8^. Thus, the epidermis of adult zebrafish, as in mice, derives from basal stem cells, further expanding the similarities of epidermal ontogeny across vertebrates ^10^.

### The importance of studying E-cadh in epidermis

E-cadh is member of a superfamily of cadherins, calcium-dependent cell-cell adhesion molecules forming junctions along the apicolateral membranes of adjacent cells ^11– 13^. E-cadh plays a key role in determining cell polarity and differentiation, and thereby in the establishment and maintenance of metazoan tissue homeostasis ^2, 14^. As epithelia are constituted by cell phenotypes with the maximum polarity and whose identity is primarily specified by E-cadh, it is key to know its expression in the epidermis establishment and maintenance ^15^. Furthermore, due to the mechano-transduction activity coupled to the acto-myosin cytoskeleton remodeling, E-cadh is involved in processes such as cell division orientation in planar polarized epithelia ^15^ and collective cell migration ^16^. Relevant as well is that E-cadh has been characterized as a potent suppressor of invasion and metastasis in epithelia ^17^, which are usually located in direct contact with mutagenic and/or carcinogenic agents responsible for 85–90 % of human cancers ^18^.

In zebrafish, E-cadh transcripts and proteins are maternally deposited. Reduced levels of the maternal and zygotic protein have been proved to delay epiboly progression with lethal phenotypes ^5^. E-cadh is required for blastomeres adhesion during the cleavage stage and later during gastrulation and epiboly ^19– 21^. Indeed, as epiboly proceeds, EVL directs cell migration and the spreading of cells of the deep cells layer (DCL) in a process that requires dynamic cell contacts remodeling mediated by E-cadh ^6^. Once the bi-layered epidermis is established, E-cadh role becomes more refined by keeping its integrity while actively remodeling cell-cell contacts within each layer. At tissue scale, this leads to cell rearrangements which establish regular geometric patterns, and loss of E-cadh results in altered epidermis topology ^5, 14, 22^. Strikingly, scarce knowledge exists regarding epidermal spatiotemporal expression of E-cadh after epiboly stages. By combining 3D-deconvolution and segmentation of AJs in epidermal cells we were able to obtain a quantitative profile of E-cadh expression during normal epidermis morphogenesis from embryonic-to-larval life of zebrafish.

## Methods

### Zebrafish husbandry

Zebrafish strain of T/AB genetic background was used as wild-type. Male and female adults of 8-months-old were obtained from the Institute of Molecular and Cellular Biology of Rosario (IBR-CONICET-UNR), Argentina, and maintained at 28°C on a 14-h light/10-h dark cycle. Adult fishes were kept in rectangular glass tanks of 12 liters at a density of (1–2 fishes/liter). In each tank, chlorine free water was constantly aerated and filtered (ATMAN hang On filter HF 0100), and renovated by 1/3 twice a week, water temperature was maintained with a heater (Atman 200W). Water pH was kept between 7.8–8.2, salinity was maintained between 350–600 TDS and nitrates were controlled using biological films included in the filtering system. Fishes were fed twice a day with dried flakes (TetraMin) and twice a week with freshly hatched artemia cysts.

After breeding, laid eggs were collected and maintained at 28°C. Then, embryos and larvae were staged according to Kimmel *et al*. ^23^. Around 20 to 25 embryos were collected at 2.5, 18, 24, 31, 48 and 72 hpf, then dechorionated and sedated with buffered tricaine methylsulfonate (MS-222, Sigma) prior to fixation. Approximately, 10–15 fixed embryos per stage were processed for immunofluorescence detection of E-cadh.

Adults and embryos were handled according to the ARRIVE guidelines and to the national guidelines from the Advisory Committee on Ethics of the Facultad de Bioquímica y Ciencias Biológicas de la Universidad Nacional del Litoral, Santa Fe, Argentina (Res. 229 and 388/2006).

### Embryos processing for immunodetection of E-cadh

All embryos were fixed *in toto* in Carnoy solution at room temperature (RT) for at least 2 h and processed according to Izaguirre *et al*. ^24^. Briefly, they were washed in PBS and permeated in 1% Triton X-100/PBS pH 7.4 for 1 h. Then, washed in PBS pH 7.4 and incubated in normal goat serum (catalogue number: S-1000 Vector Laboratories, Burlingame, CA) for 45 min, followed by overnight incubation with primary antibody anti E-cadh at 4°C, three washes in PBS, and incubation with secondary goat anti-mouse IgG-FITC antibody at RT in darkness for 2 h. Finally, they were rinsed in PBS and mounted in 50% Glycerol-PBS for microscopy imaging. Embryos directly incubated with secondary antibody and normal goat serum, were used as negative controls.

*Antibodies.* The 36/E-cadh monoclonal antibody recognizes the cytoplasmic domain of human E-cadh, regardless of phosphorylation status (clone 36 mouse IgG2a, catalogue number: 610181 Transduction Laboratories). It was diluted 1:150 and revealed with secondary goat anti-mouse IgG-FITC antibody (Sigma, catalogue number: F8771, St. Louis, MO) used at 1:100 dilution.

### Microscope settings and image acquisition

The spatial distribution of E-cadh in zebrafish epidermis was analyzed by fluorescence microscopy followed by image deconvolution and cell segmentation in 3D. The trunk was selected for the ease of orientation and image acquisition within the studied periods. Images were acquired with an inverted wide field sectioning microscope Olympus IX83 coupled to a digital camera CMOS-ORCA-Flash 2.8 (Hamamatsu), and commanded by Olympus Cell Sens software v. 1.13. Raw images were processed using FIJI v. 3.0. Sampling in xy was 0.182 µm with z-step every 0.33 µm. The epidermis was completely scanned along the trunk region. Lamp power was set at 12 %, and exposure time was experimentally determined and fixed in 370 ms, in order to avoid pixel intensity saturation and to minimize photobleaching.

### Deconvolution, intensity based segmentation of AJs and fluorescence intensity measurements

Deconvolution was applied to restore fluorescence, which improved contrast and z-resolution, enabling better definition of E-cadh in AJs for subsequent application of the 3D-segmentation tool. Quantification of E-cadh fluorescence intensity was carried throughout the epidermis bilayer (~ 6 μm) in calibrated 3D-ROIs set at 2500 µm ^2^ × 0.33 µm × 20 slices (16500 µm ^3^). First, deconvolution was performed on individual 3D-ROI by applying Richardson-Lucy algorithm ^25^ running under the open source Deconvolution Lab 2 v 2.0.0, with a theoretical point spread function ^26^. The Trainable Weka Segmentation Plugin v. 3.1.0, a classification tool based on machine learning in FIJI ^27^ was applied on each deconvolved 3D-ROI so as to create a template that would automatically find the cell boundaries by providing trainable examples of membranes and cytosol (set as background). Each segmented 3D stack was further converted into 8-bit binary 3D-mask and multiplied by the corresponding deconvolved 3D-ROI to obtain the final “Result of Classification”. On each classified image E-cadh fluorescence was quantified as the sum of pixel intensities per 3D-ROI and expressed as raw integrated density (RawIntDen). This measurement was performed on at least six 3D-ROIs per embryo to cover the trunk region, in five embryos per developmental stage. The pipeline for the image processing, theoretical psf and classifier model files are available as Supplementary File 1 as well as an example output.

### Fluorescence intensity measurements in individual EVL cells

On each classified image, 3D-ROIs of fixed volume (10 μm ^2^ × 3 μm deep) were selected along cell-cell contacts in EVL cells and fluorescence intensity was expressed as RawIntDen/cell-cell contact. To assess the fluorescence intensity in individual cells of the EVL, 3D-ROIs were manually outlined along cell perimeters to include the full membrane width and thickness and expressed as RawIntDen/cell area.

### Cell morphology and area measurements

Cell morphology and cell area were assessed in EVL and EBL cells from previously selected 3D-ROIs. Round, 4-, 5-, 6-, 7- and 8-sided cells were counted using the “polygon selection tool” in the individual layers. Mean area was expressed in the image calibrated units. Cell packing index was scored for the EVL and expressed as mean of number of cells/ ROI area (2500 μm ^2^). Area of penta- and hexagonal cells from EBL and EVL were compared for all stages ( Supplementary File 2).

### Statistical analysis

Five animals in the specified stages were obtained from three to five independent experiments. Differences in E-cadh levels between developmental stages were analyzed using a Linear Mixed Model (LMM). The assumptions of the model were checked graphically (linearity, homoscedasticity, normality of residuals and independence). The non-normality of the data was tested using the Shapiro-Wilk test. The variable “stage” was considered as fixed effects (24, 31, 48 and 72 hpf). The random effects of the model are the number of embryos per stage (5) and number of 3D-ROIs per embryo (at least 6). This number of ROIs per embryo was estimated in order to cover >90% of the embryo trunk for the selected stages.

Differences in mean cellular area and percentages for the observed polygon types were analyzed using LMM containing the same fixed and random effects but adding the variable “morphology”. The same statistical analysis was performed on the data set for the analysis of cell density and expressed as packing index (mean of number of cells / ROI area).

The Tukey’s test was used for post-hoc pair-wise comparison when an effect or an interaction was found significant. Significant differences are denoted with *p < 0.05, **p < 0.01. Data were analyzed with RStudio software’s version 1.1.453 and plotted with the BoxPlotR application or InfoStat software version 2018.

## Results

### E-cadh expression during embryonic epidermis morphogenesis

**Figure 1.**
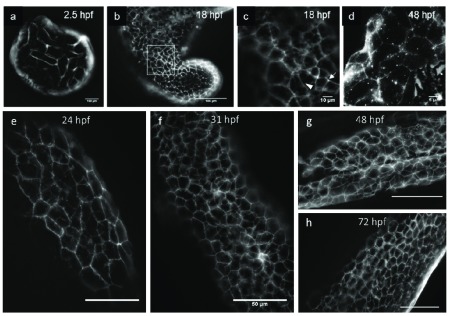
E-cadh immunofluorescence detection in zebrafish embryo epidermis. **a**) at 2.5 hours post fertilization (hpf) **b**) at 18 hpf; **c**) Zoom of selection in **b**) showing a single *puncta adherens* in contacting EVL cells (arrow) and weak E-cadh detection in the EBL (arrowhead); **d**, **g**) at 48 hpf, showing cytoplasmic dot labeling in EVL cells ; **e**– **h**) 24 to 72 hpf embryos, displaying E-cadh distribution in trunk, clearly visible in underlying EBL cells from 24 hpf. Images are contrast enhanced maximum intensity projections of 20 optical slices, z-step: 0.33 µm, scale bar: 50 µm. Objective: UPLFLN 40X 1.3 NA oil.

E-cadh expression pattern was determined in wild type zebrafish during epidermis development from 2.5 (blastula period) to 72 hpf. E-cadh protein was clearly detected in embryos at the blastula stage on epiblast cells (EVL) ( Figure 1a). By 18 hpf, during primary organogenesis both epidermis layers are already established. At this stage, E-cadh was observed in AJs in EVL cells and weakly detected in EBL cells ( Figure 1b–c). At later stages E-cadh labeling was observed as well as cytoplasmic dots, presumably in endocytic vesicles ( Figure 1d,g).

In embryos at 24 hpf, E-cadh labeling was observed in vertices ( *puncta adherens*) as well as in micro-clusters along lateral cell-cell contacts in the EVL ( Figure 1e). At this stage the underlying EBL was barely visible, detected as a faint E-cadh immunolabeling. From 31 hpf onwards, fluorescence in the underlying EBL cells was clearly detected ( Figure 1 f–h).

During the transition from embryo to larval stages the growing detection of E-cadh along cell-cell contacts parallels noticeable changes in cell size and morphology in the epidermis bilayer ( Figure 1, e–h), which were further analyzed.

**Figure 2.**
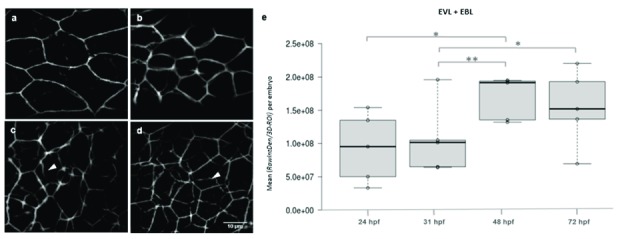
Expression patterns of E-cadherin (E-cadh) during epidermis development from stage 24-to-72 hours post fertilization (hpf). **a**) 24 hpf and **b**) 31 hpf, with incipient E-cadh labeling of the underlying epidermis basal layer (EBL) cells; **c**) 48 hpf; **d**) 72 hpf, with stronger detection of E-cadh in the EBL (arrow heads). Panels are representative 3D-ROIs classified images shown as sum of intensities in projections of 20-optical section stacks covering the epidermis thickness (6.6 μm); **e**) Box-plot of means of RawIntDen/ROI per embryo. Objective, 40X, NA 1.3 oil. Statistical significances, ** p<0.01, * p<0.05.

E-cadh levels were compared in the epidermis layers between stages 24, 31, 48 and 72 hpf, a period during which intense morphogenetic events lead to hatching and significant physiological changes occur for the resulting larvae epidermis to adapt to the aquatic environment. By implementing a 3D-Segmentation algorithm based on machine learning we were able to generate a mask to extract fluorescence intensity values along cell-cell contacts on 3D-ROIs covering the trunk epidermis bilayer and at each developmental stage ( Figure 2a–d). With this approach we measured a significant increase of E-cadh levels between 31 and 48 hpf, consistent with the visible detection of E-cadh in the EBL and the visible increase in cell density in the EVL, which was subsequently quantitated.

### Cell geometry changes during embryo to larval transition

**Figure 3.**
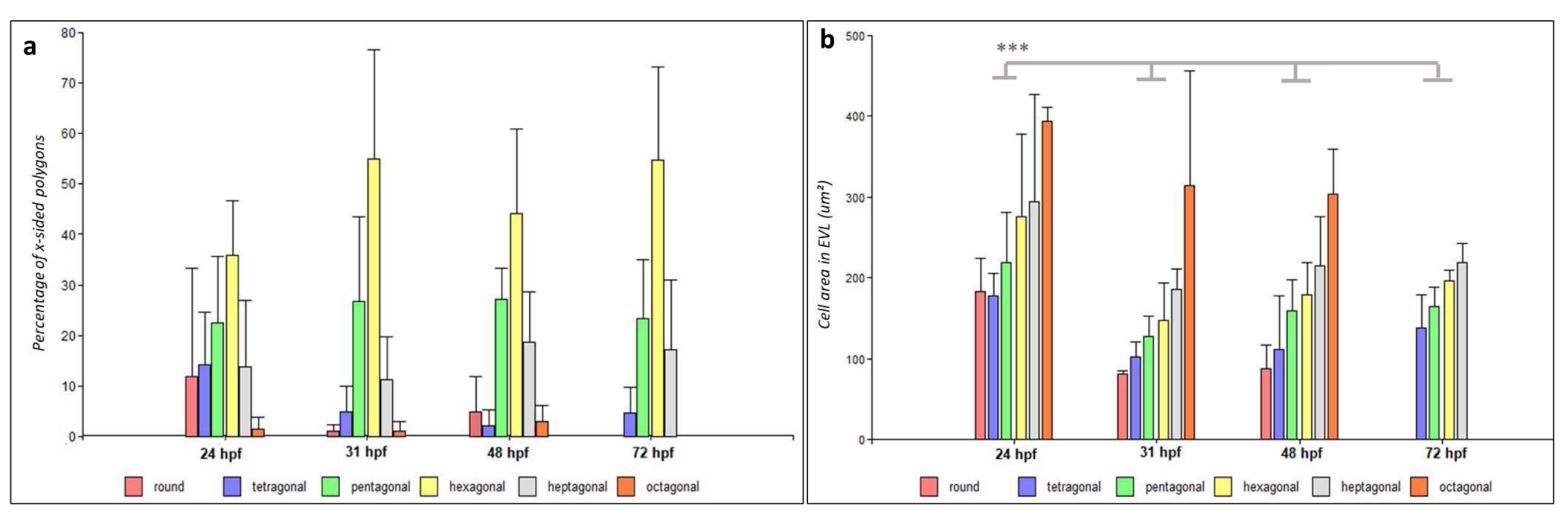
Polygon class distribution in the EVL layer from 24 to 72 hpf. **a**) Bar graph displaying mean percentages of x-sided polygons of embryos at 24 hpf (n = 81 cells), 31 hpf (n = 147 cells), 48 hpf (n = 133 cells) and 72 hpf (n = 102 cells). Hexagonal and pentagonal cells are the main cell morphologies observed in the EVL. **b**) Mean area of cell types on EVL at 24 hpf (EVL *n*= 73), 31 hpf (EVL *n*= 147), 48 hpf (EVL *n*= 133) and 72 hpf (EVL n= 102). Significant differences were found in the mean cell area for penta and hexagonal cells between stages (***p < 0.001). Data obtained from 30 3D-ROIs in five animals for each stage. The bars represent mean values ± standard deviations (SD).

Changes in cell shape, area and density were quantitated in an attempt to correlate the observed increments in E-cadh expression with cell morphology changes characteristics of developing epithelia ^28^. Similar to the other epithelia, the morphogenetic processes leading to epidermis topology development involve cell morphology changes with a predominance of hexagonal geometry in the outermost layer ^16, 24, 28^. Therefore, the distribution of the cell polygons classes was analyzed in the EVL layer. While round-cells, 4- and 8-side cell polygons were only detected in 24, 31 and 48 hpf stages and represented no more than 7, 15 and 2 % respectively, pentagonal and hexagonal shaped cells predominated in all stages ( Figure 3a). Hexagonal cells constituted approximately 34 % of EVL total cells in stage 24 hpf and 45–50% at 31, 48 and 72 hpf with a two-fold increase above pentagonal cells from 31 hpf. Hexagonal cells represented a ~ 50 % of the total cells analyzed consistent with previous reports for other species ^14^.

For all polygon types assessed in the EVL the mean cell areas decreased within the same polygon type and significantly for pentagonal and hexagonal cells from 24 to 72 hpf ( Figure 3b). In the EBL, and despite fewer cells were accessible for area measurements, this tendency was not evident ( Supplementary Figure 2). Therefore, the visible increment in cell density in the EVL was characterized by an establishment of hexagonal cell morphology.

We hypothesized then, that the significant increment of E-cadh levels in the epidermis bilayer between 31 and 48 hpf could reflect a contribution of the appearance of more cell-cell contacts per area, the addition of more protein to cell-cell contacts and the emergent detection of the protein in the underlying EBL.

**Figure 4.**
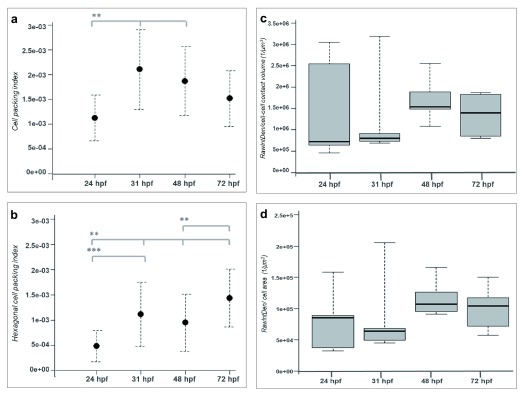
E-cadh expression profile in individual cells of the enveloping layer (EVL). **a**) Plot of means of cell packing index calculated as the average number of cells/µm ^2^; **b**) Plot of means of hexagonal cell packing index calculated as the average number of hexagonal cells/µm ^2^); **c**) Box-plot of means of RawIntDen/cell-cell contact volume; **d**) Box-plot of means of RawIntDen/average cell area. Statistical significances, ** p<0.01, ***p<0.001.

Then, to elucidate whether the increase could be due to the appearance of more cell-cell contacts per EVL area, cell density was estimated globally and for hexagonal cells, and expressed as cell packing indexes ( Figure 4a, b). Together, these results showed a significant increment in cell density in the EVL that was characterized by the establishment of hexagonal cell morphology from 24 to 72 hpf. However, there was no significant change in cell density between 31 and 48 hpf, parallel to the increment in E-cadh. 

The separation of the EVL as a sub-stack in individual 3DROIs is not completely free from the fluorescence contribution of the underlying EBL layer. Therefore, to evaluate the contribution of individual EVL cells to the observed differences in global fluorescence intensity, this was quantified in individual cell-cell contacts of fixed volume (3D-ROIs, 10 μm ^2^ × 3 μm deep) in the EVL or in individual cells. With this approach overlapping contacts with the underlying EBL cells were excluded from the measurements. Mean RawIntDen values along cell-cell contacts revealed that more E-cadh may populate individual contacts during embryonic epidermis morphogenesis and contribute to the total fluorescence increase, although differences were not significant between developmental stages ( Figure 4c). Then, fluorescence intensity was quantified in individual cells of the EVL manually outlined from the original 3D-ROIs, and expressed as RawIntDen/cell area ( Figure 4d). As expected, the expression of E-cadh in the EVL followed a similar pattern as the one obtained for the global bilayer analysis. However, differences between 31 and 48 hpf were not significant. Together this data suggested that the main contribution to the significant increase in E-cadh in the bilayer is due to the growing detection of the protein in the underlying EBL observed at 48 hpf.

Raw images for Figure 1 for 2.5 and 18 hours post fertilization (hpf). These can be viewed using FIJI or ImageJ Click here for additional data file. Copyright: © 2019 Sampedro MF et al. 2019 Data associated with the article are available under the terms of the Creative Commons Zero "No rights reserved" data waiver (CC0 1.0 Public domain dedication).

Raw and processed images of 3D-ROIs for assessing RawIntDen, cell areas and cell morphology data for 24, 31,48 and 72 hours post fertilization (hpf) for Figure 2a and Dataset 3. Raw can be viewed using FIJI or ImageJ Click here for additional data file. Copyright: © 2019 Sampedro MF et al. 2019 Data associated with the article are available under the terms of the Creative Commons Zero "No rights reserved" data waiver (CC0 1.0 Public domain dedication).

RawIntDen, cell areas and cell morphology counts for Figure 2–Figure 4 Click here for additional data file. Copyright: © 2019 Sampedro MF et al. 2019 Data associated with the article are available under the terms of the Creative Commons Zero "No rights reserved" data waiver (CC0 1.0 Public domain dedication).

## Discussion

Epithelial architecture retains essential features such as apical/basal cell polarization, formation of cell–cell junctions, and the constitution of a paracellular diffusion barrier, all of which enable epithelia to serve a great diversity of biological functions ^29^. The organization of mature epithelia into packaging of hexagonal shaped cells is a feature evolutionarily conserved ^14, 30^. This appears to be the optimum arrangement concerning the transduction of forces with minimum energy costs. The role of E-cadh in molecular architecture of epithelia has been studied extensively in animal tissues, partially owing to its decisive play in human cancers ^31^. In frog and fish embryos, as in others, cadherins are the main adhesion factors responsible for regulating the shape of the embryo and its role has been thoroughly described during epiboly and gastrulation ^32– 34^. On established animal epidermis, E-cadh performs fine-tuned cell-cell contact remodeling to maintain tissue integrity while the body axis elongates, this is characterized by modulation of cell shape, size and density to achieve the stable hexagonal arrangement ^28, 35, 36^.

In zebrafish, an immature epidermis is established at 24 hpf, formed by the enveloping layer (EVL) and the epidermal basal layer (EBL). Despite numerous descriptions about E-cadh role in epiboly and gastrulation, there is scarce information about E-cadh distribution in the epidermis beyond this stage and during the embryo to larval transition. During this period body axis elongates from 1 mm at 24 hpf to 3.5 mm at 72 hpf and embryos undergo hatching asynchronously between 48 and 60 hpf ^23^. Once in direct contact with water, the embryonic epidermis is the main protective barrier against pathogens. Therefore, we find it relevant to study the spatiotemporal distribution of E-cadh in zebrafish and elucidate a relationship between E-cadh levels, cell morphology and cell density in the epidermis bilayer from “embryos” to “larvae”.

We implemented a trainable 3D-segmentation tool in FIJI ^37^ to extract fluorescence intensity values from epithelial cells in *in toto* immunolabeled epidermis. Global expression was estimated from these 3D-segmentation volumes excluding cytoplasm (E-cadh in endosomal or in reticulo-endoplasmic compartments) in the bi-layered epidermis from embryo to larval stages. At present, only one pipeline method was reported for segmentation and tracking of epithelial cells based on the detection of the AJs in voxels in the *Drosophila notum* and leg epithelium ^38^.

At the membrane level, E-cadh protein concentrates in clusters detected as *puncta adherens* in cell vertices or as lateral micro-clusters at 24 hpf, that turns into a continuous belt structure from stage 31 hpf onwards, when intracellular E-cadh is also frequently observed in the cytoplasm of EVL cells.

Intensity based analysis showed that growing levels of E-cadh along cell-cell contacts during zebrafish epidermis development correlate with cell morphology changes towards hexagonal geometry. Specifically, within a short period between 24 and 31 hpf, a ~two-fold increase in cell density parallels the appearance of penta- and hexagonal cells together representing ~75 % of the polygons classes similarly to other animal models ^14^.

Global bilayer analysis of E-cadh fluorescence intensity revealed a significant increase in protein expression between 31 and 48 hpf. In an attempt to establish the contribution of individual layers to the observed difference, the outermost EVL layer was analyzed by selecting individual cells or cell-cell contacts without the interference of the fluorescence coming from the basal layer.

Mean E-cadh levels measured in fixed cell-cell contact volumes of EVL cells showed a steady increase from 24 to 72 hpf, but without significant differences between stages. When E-cadh levels were estimated per average cell area in the EVL, a visible increase was observed between 31 and 48 hpf, although non-significant, indicating that the emergent detection of E cadh in the EBL from 31 hpf onwards may indeed contribute to the significant increase in the protein levels measured in the epidermis bilayer.

We cannot overlook that during this embryonic period characterized body elongation, either cell proliferation or adherens junctions remodeling, through active E-cadh trafficking could account for the observed increase in hexagonal cell density in the epidermis ^39, 40^. These mechanisms has been well described for the hexagonal cell packing in the developing *D. melanogaster* pupal wing under polarized trafficking of E-cadh ^28^ and should be further analyzed *in vivo* within this period in zebrafish.

As a novel outcome, a recent report proposed that tension generated by the E-cadh/AmotL2/actin filaments complexes plays a crucial role in developmental processes such as epithelial geometrical packing as well as generation of forces required for blastocyst hatching both in mouse and human ^35^. In zebrafish, the hatching process is the result of combined enzymatic digestion of the chorion ^41^ together with mechanical forces that drive the embryo out of the yolk sac in a similar way as observed for mouse and human blastocyst hatching. In the present work, a peak in E-cadh membrane level together with an increase in hexagonal cells density in the enveloping layer were detected in the epidermis around the time of spontaneous hatching. Therefore, it is conceivable, that cell morphology remodeling leading to hexagonally packed geometry followed by a significant increase in E cadh may achieve high cell surface compactness and stiffness of the epidermis required for efficient mechanical disruption of the chorion.

## Conclusions

The presented results show that during the establishment of embryonic epidermis in zebrafish, growing level of E-cadh protein correlates with increased density of hexagonal cells in the EVL and the detection of adherens junctions in cell-cell contacts in the EBL, significantly between 31 and 48 hpf. This differentiated and compact epidermal tissue is most likely to support mechanical stress prior to hatching which starts around 48 hpf, when the embryo contacts for the first time directly the aquatic environment.

The combination of classical immunofluorescence, image deconvolution with intensity based segmentation in 3D offers a powerful tool to study the spatial arrangement of cell-cell adhesion proteins and cell morphology in bi-layered epithelia that can be applied to cadherin morphants, to other species or processes such as wound healing and re-epithelialization of the skin.

## Data availability

The data referenced by this article are under copyright with the following copyright statement: Copyright: © 2019 Sampedro MF et al.

Data associated with the article are available under the terms of the Creative Commons Zero "No rights reserved" data waiver (CC0 1.0 Public domain dedication). 

Dataset 1: Raw images for Figure 1 for 2.5 and 18 hours post fertilization (hpf). These can be viewed using FIJI or ImageJ 10.5256/f1000research.15932.d217819 ^42^

Dataset 2: Raw and processed images of 3D-ROIs for assessing RawIntDen, cell areas and cell morphology data for 24, 31,48 and 72 hours post fertilization (hpf) for Figure 2a and Dataset 3. Raw can be viewed using FIJI or ImageJ 10.5256/f1000research.15932.d217820 ^43^

Dataset 3: RawIntDen, cell areas and cell morphology counts for Figure 2– Figure 4 https://doi.org/10.5256/f1000research.15932.d236505 ^44^

## Supplementary material

Supplementary File 1: Image processing pipeline for E cadherin fluorescence intensity segmentation and quantitation in 3D-ROIs selected in the trunk epidermis

Supplementary File 2: theoretical psf stack for image processing pipeline

Supplementary Figure 2: Mean cell area of the main cellular types of the enveloping layer (EVL) and epidermal basal layer (EBL) of Danio rerio from 24 to 72 hours post fertilization (hpf) (EVL n=48; EBL n=21), 31 hpf (EVL n=108; EBL n=30), 48 hpf (EVL n=101; EBL n=47) and 72 hpf (EVL n=79; EBL n=45)

## References

[1] Cauna N: The free penicillate nerve endings of the human hairy skin. *J Anat.* 1973;115(Pt 2):277–88. 4756249 PMC1271512

[2] Muroyama A Lechler T: Polarity and stratification of the epidermis. *Semin Cell Dev Biol.* 2012;23(8):890–896. 10.1016/j.semcdb.2012.08.008 22960184 PMC3549552

[3] Warga RM Nüsslein-Volhard C: Origin and development of the zebrafish endoderm. *Development.* 1999;126(4):827–838. 9895329 10.1242/dev.126.4.827

[4] Chang WJ Hwang PP: Development of zebrafish epidermis. *Birth Defects Res C Embryo Today.* 2011;93(3):205–214. 10.1002/bdrc.20215 21932430

[5] Slanchev K Carney TJ Stemmler MP: The epithelial cell adhesion molecule EpCAM is required for epithelial morphogenesis and integrity during zebrafish epiboly and skin development. *PLoS Genet.* 2009;5(7):e1000563. 10.1371/journal.pgen.1000563 19609345 PMC2700972

[6] Reig G Cerda M Sepúlveda N: Extra-embryonic tissue spreading directs early embryo morphogenesis in killifish. *Nat Commun.* 2017;8: 15431. 10.1038/ncomms15431 28580937 PMC5465322

[7] Little SC Mullins MC: Extracellular modulation of BMP activity in patterning the dorsoventral axis. *Birth Defects Res Part C Embryo Today Rev.* 2006;78(3):224–242. 10.1002/bdrc.20079 17061292

[8] Le Guellec D Morvan-Dubois G Sire JY: Skin development in bony fish with particular emphasis on collagen deposition in the dermis of the zebrafish (Danio rerio). *Int J Dev Biol.* 2003;48(2–3):217–231. 15272388 10.1387/ijdb.15272388

[9] Cline A Feldman SR: Zebrafish for modeling skin disorders. *Dermatol Online J.* 2016;22(8): pii: 13030/qt4ws351w8. 27617951

[10] Lee RT Asharani PV Carney TJ: Basal keratinocytes contribute to all strata of the adult zebrafish epidermis. Hogan B, editor. *PLoS One.* 2014;9(1):e84858. 10.1371/journal.pone.0084858 24400120 PMC3882266

[11] Hulpiau P van Roy F: Molecular evolution of the cadherin superfamily. *Int J Biochem Cell Biol.* 2009;41(2):349–369. 10.1016/j.biocel.2008.09.027 18848899

[12] Yoshida C Takeichi M: Teratocarcinoma cell adhesion: identification of a cell-surface protein involved in calcium-dependent cell aggregation. *Cell.* 1982;28(2):217–224. 10.1016/0092-8674(82)90339-7 7060128

[13] Harris TJ Tepass U: Adherens junctions: from molecules to morphogenesis. *Nat Rev Mol Cell Biol.* 2010;11(7):502–14. 10.1038/nrm2927 20571587

[14] Gibson WT Gibson MC: Cell topology, geometry, and morphogenesis in proliferating epithelia. *Curr Top Dev Biol.*Elsevier;2009;89:87–114. 10.1016/S0070-2153(09)89004-2 19737643

[15] Ragkousi K Gibson MC: Cell division and the maintenance of epithelial order. *J Cell Biol.* 2014;207(2):181–188. 10.1083/jcb.201408044 25349258 PMC4210436

[16] Theveneau E Mayor R: Collective cell migration of epithelial and mesenchymal cells. *Cell Mol Life Sci.* 2013;70(19):3481–3492. 10.1007/s00018-012-1251-7 23314710 PMC11113167

[17] Petrova YI Schecterson L Gumbiner BM: Roles for E-cadherin cell surface regulation in cancer. *Mol Biol Cell.* 2016;27(21):3233–3244. 10.1091/mbc.E16-01-0058 27582386 PMC5170857

[18] Izaguirre MF Casco VH: E-cadherin roles in animal biology: A perspective on thyroid hormone-influence. *Cell Commun Signal.* 2016;14(1):27. 10.1186/s12964-016-0150-1 27814736 PMC5097364

[19] Babb SG Marrs JA: E-cadherin regulates cell movements and tissue formation in early zebrafish embryos. *Dev Dyn.* 2004;230(2):263–77. 10.1002/dvdy.20057 15162505

[20] Kane DA Hammerschmidt M Mullins MC: The zebrafish epiboly mutants. *Development.* 1996;123:47–55. 9007228 10.1242/dev.123.1.47

[21] Kane DA McFarland KN Warga RM: Mutations in *half baked*/E-cadherin block cell behaviors that are necessary for teleost epiboly. *Development.* 2005;132(5):1105–16. 10.1242/dev.01668 15689372

[22] Sonawane M Martin-Maischein H Schwarz H: Lgl2 and E-cadherin act antagonistically to regulate hemidesmosome formation during epidermal development in zebrafish. *Development.* 2009;136(8):1231–1240. 10.1242/dev.032508 19261700

[23] Kimmel CB Ballard WW Kimmel SR: Stages of embryonic development of the zebrafish. *Dev Dyn.* 1995;203(3):253–310. 10.1002/aja.1002030302 8589427

[24] Izaguirre MF Larrea D Adur JF: Role of E-cadherin in epithelial architecture maintenance. *Cell Commun Adhes.* 2010;17(1):1–12. 10.3109/15419061003686938 20353345

[25] Fish DA Brinicombe AM Pike ER: Blind deconvolution by means of the Richardson-Lucy algorithm. *J Opt Soc Am A.* 1995;12(1):58–65. 10.1364/JOSAA.12.000058

[26] Sage D Donati L Soulez F: DeconvolutionLab2: An open-source software for deconvolution microscopy. *Methods.* 2017;115:28–41. 10.1016/j.ymeth.2016.12.015 28057586

[27] Arganda-Carreras I Kaynig V Rueden C: Trainable Weka Segmentation: a machine learning tool for microscopy pixel classification. *Bioinformatics.* 2017;33(15):2424–2426. 10.1093/bioinformatics/btx180 28369169

[28] Classen AK Anderson KI Marois E: Hexagonal Packing of Drosophila Wing Epithelial Cells by the Planar Cell Polarity Pathway. *Dev Cell.* 2005;9(6):805–817. 10.1016/j.devcel.2005.10.016 16326392

[29] Tunggal JA Helfrich I Schmitz A: E-cadherin is essential for *in vivo* epidermal barrier function by regulating tight junctions. *EMBO J.* 2005;24(6):1146–1156. 10.1038/sj.emboj.7600605 15775979 PMC556407

[30] Li Y Naveed H Kachalo S: Mechanisms of Regulating Cell Topology in Proliferating Epithelia: Impact of Division Plane, Mechanical Forces, and Cell Memory.Connon CJ, editor. *PLoS One.* 2012;7(8):e43108. 10.1371/journal.pone.0043108 22912800 PMC3422310

[31] Jeanes A Gottardi CJ Yap AS: Cadherins and cancer: how does cadherin dysfunction promote tumor progression? *Oncogene.* 2008;27(55):6920–6929. 10.1038/onc.2008.343 19029934 PMC2745643

[32] Zalik SE Lewandowski E Kam Z: Cell adhesion and the actin cytoskeleton of the enveloping layer in the zebrafish embryo during epiboly. *Biochem Cell Biol.* 1999;77(6):527–542. 10.1139/bcb-77-6-527 10668630

[33] Bruce AE: Zebrafish epiboly: Spreading thin over the yolk. *Dev Dyn.* 2016;245(3):244–258. 10.1002/dvdy.24353 26434660

[34] Schepis A Nelson WJ: Adherens junction function and regulation during zebrafish gastrulation. *Cell Adh Migr.* 2012;6(3):173–8. 10.4161/cam.20583 22568981 PMC3427231

[35] Hildebrand S Hultin S Subramani A: The E-cadherin/AmotL2 complex organizes actin filaments required for epithelial hexagonal packing and blastocyst hatching. *Sci Rep.* 2017;7(1): 9540. 10.1038/s41598-017-10102-w 28842668 PMC5572699

[36] Lecuit T Lenne PF: Cell surface mechanics and the control of cell shape, tissue patterns and morphogenesis. *Nat Rev Mol Cell Biol.* 2007;8(8):633–44. 10.1038/nrm2222 17643125

[37] Arganda-Carreras I Kaynig V Rueden C: Trainable_ Segmentation: Release v3.1.2. *zenodo.* 2016 10.5281/zenodo.59290

[38] Cilla R Mechery V Hernandez de Madrid B: Segmentation and tracking of adherens junctions in 3D for the analysis of epithelial tissue morphogenesis. *PLoS Comput Biol.* 2015;11(4):e1004124. 10.1371/journal.pcbi.1004124 25884654 PMC4401792

[39] Le TL Yap AS Stow JL: Recycling of E-cadherin: a potential mechanism for regulating cadherin dynamics. *J Cell Biol.* 1999;146(1):219–232. 10.1083/jcb.146.1.219 10402472 PMC2199726

[40] West JJ Harris TJ: Cadherin Trafficking for Tissue Morphogenesis: Control and Consequences. *Traffic.* 2016;17(12):1233–1243. 10.1111/tra.12407 27105637

[41] Yamagami K: Mechanisms of hatching in fish: secretion of hatching enzyme and enzymatic choriolysis. *Am Zool.* 1981;21(2):459–471. 10.1093/icb/21.2.459

[42] Sampedro MF Izaguirre MF Sigot V: Dataset 1 in: E-cadherin expression pattern during zebrafish embryonic epidermis development. *F1000Research.* 2018 10.5256/f1000research.15932.d217819 PMC6234749 30473778

[43] Sampedro MF Izaguirre MF Sigot V: Dataset 2 in: E-cadherin expression pattern during zebrafish embryonic epidermis development. *F1000Research.* 2018 10.5256/f1000research.15932.d217820 PMC6234749 30473778

[44] Sampedro MF Izaguirre MF Sigot V: Dataset 3 in: E-cadherin expression pattern during zebrafish embryonic epidermis development. *F1000Research.* 2018 10.5256/f1000research.15932.d236505 PMC6234749 30473778

