# Supplementary material for: E-cadherin expression pattern during zebrafish embryonic epidermis development
Source: F1000Res. 2019 Feb 18;7:1489. Originally published 2018 Sep 18. [Version 3] doi: 10.12688/f1000research.15932.3 (PMC6234749; doi:10.12688/f1000research.15932.3)
Supplement: Supplementary file 4 [file f1000research-7-20004-s0003.tgz › 57932a88-a7cd-4d52-9a35-9a3871c250b8_Image_processing__for_Ecadh_fluorescence_measurements_from_dataset_2.docx]

Sampedro et al.

**Supplementary Information**

**Image processing pipeline for E cadherin fluorescence intensity segmentation and quantitation in 3D-ROIs selected in the trunk epidermis**

**Quantitation of E-cadherin immunolabeling**

1. Select six 3D-ROIs for each embryo and set volume: 2500 µm^2^ x 0,33 µm x 20 slices (16500 µm^3^).
2. Load 3D-ROIs raw images 16 bits
3. Open plug-in Deconvolution Lab 2 v 2.0.0 in FIJI 3.0
4. Select 3D-ROI and theoretical psf stack (40X 16 bits, psf Icy, provided as additional file)
5. Apply Deconvolution Richardson-Lucy algorithm with 10 iterations
6. Image: Result of RL save as tiff (32 bits).The restored image obtained as 32 bits is again linearly stretched to 16 bits image
7. Open plugin Trainable Weka Segmentation 3D v 3.1.0
8. Load Result of RL 16 bits (it does not work with 32 bits images)
9. Load classifier model Random- Forest (The classifier tool was previously trained defining to classes (class 1: membranes, class 2: cytosol area), and saved as classifier-model (the model used was provided as an additional file)
10. Apply classifier. Resulting image is colour image 8 bits. Save this image as Classified Result of RL.
11. Duplicate and convert Classified Result of RL (8-bit colour) to 8-bit, then select Process/Binary mask to set 0 background (cytoplasm) and 255 values for cell membranes.
12. Go to Process/Math/Divide image by 255.
13. Multiply Classified image binary obtained in l) by Result of RL 16 bits and save as “Result of Classification”16 bits tiff.
14. For each “Result of Classification” of ROI go to Stack/z projection/Sum of slices
15. On the resulting image “Sum of slices”, go to Analyze/Measure “Raw Integrated Density” (RawIntDen) which is the sum of the values of the pixels in the “3D classified image”


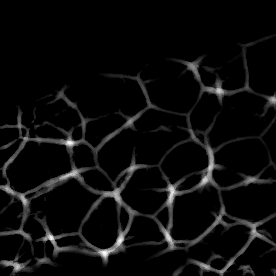

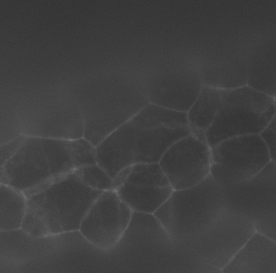

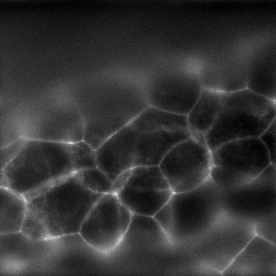


a

b

c

**Figure S1:** Image processing a) single slice from original image, acquired at very low excitation intensity to minimize photobleaching, b) same image in a) after deconvolution, (c) Image obtained in step 14 “Sum of slices” obtained from the projection of the 3D classified image. The value “Raw Integrated Density” obtained from this image represents the sum of intensities for all the pixels, which was then used as a measurement of E cadherin levels in each ROI 3D.
