# Supplementary figures and images for: E-cadherin expression pattern during zebrafish embryonic epidermis development
Source: F1000Res. 2019 Feb 18;7:1489. Originally published 2018 Sep 18. [Version 3] doi: 10.12688/f1000research.15932.3 (PMC6234749; doi:10.12688/f1000research.15932.3)

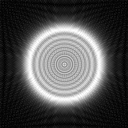

Supplement: Supplementary file 5 [file f1000research-7-20004-s0005.tgz › a6df2029-8bec-4a95-9e3e-c59fb06a4155_psf_40X_fitc_for_RL_deconvolution.tif]

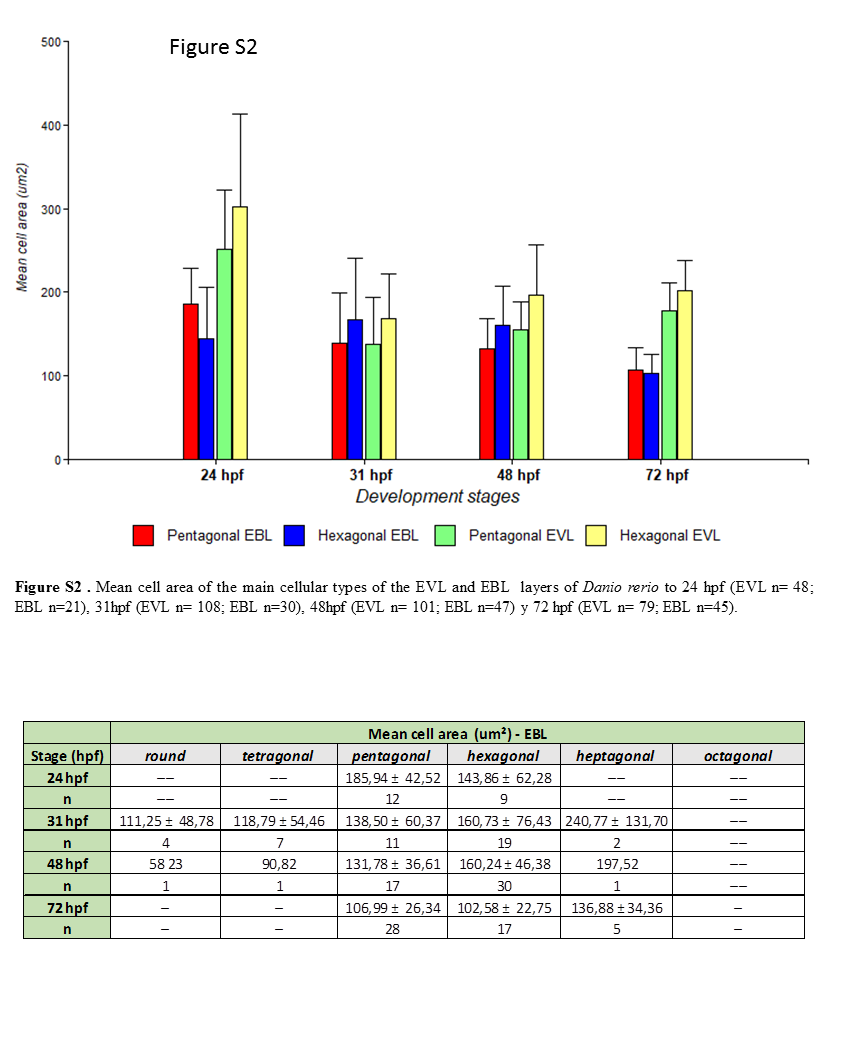

Supplement: Supplementary file 6 [file f1000research-7-20004-s0004.tgz › e046a96a-8c4c-4b34-bcb8-860de8378630_Figure_S2_Cell_morphology_EBL.tif]
