# Supplementary material for: E-cadherin expression pattern during zebrafish embryonic epidermis development
Source: F1000Res. 2019 Feb 18;7:1489. Originally published 2018 Sep 18. [Version 3] doi: 10.12688/f1000research.15932.3 (PMC6234749; doi:10.12688/f1000research.15932.3)
Supplement: Peer review history

# Peer review: Reviewer response for version 3

**DOI:** 10.5256/f1000research.20004.r44632

**Published:** 2019-02-28

**Authors:** Tony J. C. Harris

**Affiliations:** 1 Department of Cell & Systems Biology, University of Toronto, Toronto, ON, Canada

The authors have addressed my remaining concern.

I have read this submission. I believe that I have an appropriate level of expertise to confirm that it is of an acceptable scientific standard.

# Peer review: Reviewer response for version 3

**DOI:** 10.5256/f1000research.20004.r44631

**Published:** 2019-02-25

**Authors:** James A. Marrs

**Affiliations:** 1 Department of Biology, Indiana University-Purdue University Indianapolis, Indianapolis, IN, USA

The revised manuscript improved the overall message and has addressed my original concerns.

I have read this submission. I believe that I have an appropriate level of expertise to confirm that it is of an acceptable scientific standard.

# Peer review: Reviewer response for version 2

**DOI:** 10.5256/f1000research.19077.r41922

**Published:** 2018-12-27

**Authors:** Tony J. C. Harris

**Affiliations:** 1 Department of Cell & Systems Biology, University of Toronto, Toronto, ON, Canada

The authors' provide reasonable responses and revisions for many of my past concerns.

 A remaining concern is that for both graphs of Fig 3 (Polygon class distribution in the EVL layer from 24 to 72 hpf), "No significant differences were found between stages". Because significance cannot be shown, statements about increased frequency of hexagonal cells from 24 to 72 hpf do not seem to be supported by the current data. The statements should be revised or more data should be collected.

I have read this submission. I believe that I have an appropriate level of expertise to confirm that it is of an acceptable scientific standard, however I have significant reservations, as outlined above.

# Response

**Published:** 2019-02-01

**Authors:** Valeria Sigot

**Affiliations:** Universidad Nacional de Entre Ríos, Argentina

According to your observations and following your suggestions we collected additional data for the EVL at 24 hpf. We performed the immunofluorescence protocol in another embryo fixed at 24 hpf. Again, there was no significant increase in the frequencies of hexagonal cells/stage from 24 to 72 hpf at the EVL ( **p=0.1514**).Therefore, figure 3a will remain the same.

 % (percentage of polygon classes)

             numDF denDF  F-value p-value

(Intercept)      1    92 350.1999  <.0001

stage            3    92   0.0000  1.0000

morpho           5    92  65.5217  <.0001

stage:morpho    15    92   1.4270 **0.1514**

Then, with this additional data we analyzed whether there was a difference in the number of hexagonal cells/µm ^2^ between stages (30 ROis of 2500 um2 in 5-6 embryos per stage). We carried out the same analysis applied for the global cell packing analysis (Fig 4b in the published version) but only for hexagonal cells of the EVL.  

After running the LMM method in R, a significant increase in the average number of hexagonal cells/ µm ^2 ^from 24 to 72 hpf in the EVL was revealed and because the total epidermis area analyzed was the same for all stages this indicates as well a significant increase in the absolute number of hexagonal cells from 24 to 72 hpf. However, and in agreement with the global cell packing the difference was not significant between 31 and 48 hpf.

  

We will provide a plot of means of hexagonal cell packing index calculated as the average number of hexagonal cells/µm ^2^) as a new panel in figure 4 and the corresponding dataset and analysis in R software.

Then, we analyze again the data from Figure 3b with the Lineal Mixed model in R and found out that indeed there was a significant decrease in cell area for penta- and hexagonal cells between stages (table below).which by mistake was not detected in the previous analysis, despite the visibly differences in the sd

We will provide the new analysis in R for the same datasets and we will add the asterisks to denote the statistical significance of the differences in panel b.

Linear Hypotheses:

             Estimate Std. Error z value Pr(>|z|)    

31 - 24 == 0 -195.509     16.570 -11.799   <0.001 ***

48 - 24 == 0 -198.963     16.124 -12.340   <0.001 ***

72 - 24 == 0 -158.279     19.769  -8.006   <0.001 ***

48 - 31 == 0   -3.454     12.826  -0.269   0.9930    

72 - 31 == 0   37.231     16.619   2.240   0.1100    

72 - 48 == 0   40.684     16.956   2.399   0.0751 .  

---

Signif. codes:  0 ‘***’ 0.001 ‘**’ 0.01 ‘*’ 0.05 ‘.’ 0.1 ‘ ’ 1

(Adjusted p values reported -- single-step method)

 Therefore, a significant increase in the number of hexagonal cells correlates now with a significant decrease in hexagonal cell area indicating the ordering towards hexagonal cell packing during epidermis morphogenesis.

# Peer review: Reviewer response for version 2

**DOI:** 10.5256/f1000research.19077.r41921

**Published:** 2018-12-20

**Authors:** Marc Muller

**Affiliations:** 1 Laboratory for Organogenesis and Regeneration, GIGA-Research, University of Liège, Liège, Belgium

The paper clearly shows that the implemented approach is able to provide new insights into the evolution of epithelial cell layers. 

 Comments to version 1 have been addressed in the response, maybe some of this response should end up in the discussion part in the main text.

I have read this submission. I believe that I have an appropriate level of expertise to confirm that it is of an acceptable scientific standard.

# Peer review: Reviewer response for version 1

**DOI:** 10.5256/f1000research.17400.r40062

**Published:** 2018-11-13

**Authors:** James A. Marrs

**Affiliations:** 1 Department of Biology, Indiana University-Purdue University Indianapolis, Indianapolis, IN, USA

The manuscript by Sampedro et al. provides an interesting and useful contribution to epidermis development in the zebrafish. Cadherin adhesion is among the mechanisms that control epidermis development, and the authors contribute an initial description of cadherin adhesion molecule expression changes during epidermis maturation. The image analysis and cell shape changes observed will provide a starting point for additional studies. However, I have some concerns about the manuscript.

1. The monoclonal antibody that the authors used to examine E-cadherin was raised to human E-cadherin cytoplasmic domain sequences. This antibody cross reacts with cadherins in various species and with cadherin types other than E-cadherin. E-cadherin is probably the most prevalent cadherin in the epidermis but this was not shown.  If other cadherins are expressed, then this antibody is likely to detect those cadherins too.
2. Flurorescence microscopy can be a useful way to measure protein expression, but several controls would be necessary to ensure that expression is being detected within the linear range of flurorescence detection. These controls were not evident, and expression was not validated with other approaches.
3. The authors state that the prospective epidermis is established at 24 hpf, but this prospective structure is established at neurulation, which occurs significantly earlier.

Overall, this is a useful contribution to our understanding of cadherin adhesion activity during epidermis development.

I have read this submission. I believe that I have an appropriate level of expertise to confirm that it is of an acceptable scientific standard.

# Response

**Published:** 2018-12-05

**Authors:** Valeria Sigot

**Affiliations:** Universidad Nacional de Entre Ríos, Argentina

**1-The monoclonal antibody that the authors used to examine E-cadherin was raised to human E-cadherin cytoplasmic domain sequences. This antibody cross reacts with cadherins in various species and with cadherin types other than E-cadherin. E-cadherin is probably the most prevalent cadherin in the epidermis but this was not shown.  If other cadherins are expressed, then this antibody is likely to detect those cadherins too.**

 VS: We are aware of this, indeed the supplier states in the datasheet that this antibody has some degree of cross-reactivity to P-Cadherin. This cadherin is expected to be expressed in the skin, its presence has been reported in the basal layer of the epidermis in mice most prominently in hair follicles (Tinkle et al. PNAS 2008 105 (40) 15405-15410; https://doi.org/10.1073/pnas.0807374105).

 In adult zebrafish, the presence of P-cadherin was screened through immunohistochemistry in regenerating blastema but without positive detection (Mateus et al.Development (2015) 142, 2752-2763 doi:10.1242/dev.119701.

 We did test an anti N cadherin, Clone 32/N-Cadherin (cat.  610920 BD Transduction Lab) IgG1 and **we **observed only a dim membrane labeling in the eye (figure below).

**2-Fluorescence microscopy can be a useful way to measure protein expression, but several controls would be necessary to ensure that expression is being detected within the linear range of fluorescence detection. These controls were not evident, and expression was not validated with other approaches.**

 VS: We understand your concern and we are aware of the limitations that quantitative microscopy has, so far our intention was to be accurate in measuring the change in protein expression than the amount itself of the protein at a particular stage.In the “methods” section we stated that all the acquisition settings were held equal for each 3D stack, but we will specify in the new version of the manuscript that lamp power was set at 6% and the exposure time set at 370 msec. With these settings we collected z-stacks with lower than desired contrast and counted on the improvement in SNR after applying the deconvolution algorithm. Under these conditions photo-bleaching of FITC could be neglected and the distribution of the available intensity levels for all embryonic development stages corresponded well to the linear dynamic range of detection of the CMOS-ORCA-Flash 2.8 (4500:1, calculated from the ratio of the full well capacity and readout noise).With our approach we aim to implement an image processing routine for extracting fluorescence intensity and cell morphology features in a bilayer with a single immunofluorescence and acquisition procedure, which can be applied as well to living embryos using a transgenic reporter protein in laboratories with limited equipment and resources.

**3-The authors state that the prospective epidermis is established at 24 hpf, but this prospective structure is established at neurulation, which occurs significantly earlier.**

 VS: Certainly, we misused the term “prospective” to refer to the immature bilayered epidermis this will be corrected in the manuscript

# Peer review: Reviewer response for version 1

**DOI:** 10.5256/f1000research.17400.r39783

**Published:** 2018-11-12

**Authors:** Marc Muller

**Affiliations:** 1 Laboratory for Organogenesis and Regeneration, GIGA-Research, University of Liège, Liège, Belgium

This paper represents a thorough microscopic analysis of E-Cadh expression, as detected by immunostaining, during zebrafish development in the two layers of epidermal cells.

- Fig. 1: what does the white arrow in Fig. 1c point to? Also, weak detection in EBL cells is not so obvious at 18hpf, maybe another arrow (head) would be helpful.
- Fig. 2: Comparison of E-cadh levels between stages: how far can it be excluded that immunodetection by the antibodies may be systematically different at different stages, considering the important changes in the epidermal cells, especially between 31 and 48hpf (hatching)?
- This comment also applies to Fig. 4, where in addition the differences between stages are either non-significant or dismissed by the authors. 

Overall, this comment sheds some doubt onto the comparisons of intensities between stages, while the changes in topology (polygon classes) is more secure. I was wondering whether counting of the *puncta adherens*, independent of intensity, could be performed to further support the conclusions. Also, the weaker labelling of the deeper EBL layer would be further supported by performing immunodetection on sections.

I have read this submission. I believe that I have an appropriate level of expertise to confirm that it is of an acceptable scientific standard, however I have significant reservations, as outlined above.

# Response

**Published:** 2018-11-28

**Authors:** Valeria Sigot

**Affiliations:** Universidad Nacional de Entre Ríos, Argentina

- Fig. 1: what does the white arrow in Fig. 1c point to? Also, weak detection in EBL cells is not so obvious at 18hpf, maybe another arrow (head) would be helpful.

***VS: The white arrow indicates a single puncta adherens in contacting EVL cells. Following your suggestion, an arrowhead will point the weak detection in EBL cells at 18 hpf.***

 Fig. 2: Comparison of E-cadh levels between stages: how far can it be excluded that immunodetection by the antibodies may be systematically different at different stages, considering the important changes in the epidermal cells, especially between 31 and 48hpf (hatching)? This comment also applies to Fig. 4, where in addition the differences between stages are either non-significant or dismissed by the authors. 

***VS: If we understood your question, your concern refers to how we can explain differences in E-cadh expression whilst cells are changing in size and shape so distinctly between stages and in particular between 31 and 48 hpf.***

***Actually, between 31 and 48 hpf there is no significant increase in cell density in the EVL (Fig 4b), Thus, we exclude that the appearance of more cell-cell contacts per area (decrease in cell size and increase in the percentage of hexagonal cells) is the main contribution to the observed difference in E-cadh expression in the EVL. This is explained in the text, but probably the asterisks are misleading in figure 4b, (we will correct that in the new version).***

***The graphs in Figure 4 a and c clearly show this "trend" in the increase in the expression level from 31 to 48 hpf, either by quantifying cadherin along fixed volumes of cell-cell contacts, or in individual cells of the EVL. However, this increment was not enough to account for the significant difference observed globally (EVL+EBL) thus we conclude that is the growing detection of E-cadh at the EBL which contributes to this difference between 31 and 48 hpf.***

***We did not dismiss the non-significant results shown in Figure 4 which are only for EVL. The Linear Mixed Model approach proved robust enough for our analysis based on intensity information.***

 Overall, this comment sheds some doubt onto the comparisons of intensities between stages, while the changes in topology (polygon classes) is more secure. I was wondering whether counting of the *puncta adherens*, independent of intensity, could be performed to further support the conclusions.

***We understand your concern and we are aware of the limitations that quantitative microscopy has, so far our intention was to be more accurate in measuring the change in protein expression than the amount itself of the protein at a particular stage.  Even though, as you propose it is more secure to extract morphological data or counting puncta adherens, our approach supported by the LMM statistical method, allows analysing larger areas of epidermis being more representative and descriptive of the tissue.***

 Also, the weaker labelling of the deeper EBL layer would be further supported by performing immunodetection on sections.

**It is true, but we prefer to take advantage of the in-toto labeling and imaging of the whole mounted embryos maintaining as much as possible an intact epidermis. In addition, as *we do not have the resources to label specifically the basal cells for instance with an anti p63 antibody, we used this differential analysis of the global (EVL+EBL) and the EVL to account for the contribution of the E-cadh expression from the EBL.***

***With our approach we aim to implement an image processing routine for extracting fluorescence intensity and cell morphology features in a bilayer with a single immunofluorescence and acquisition procedure, which can be applied as well for live embryos using a transgenic reporter protein in laboratories with limited equipment and resources.***

# Peer review: Reviewer response for version 1

**DOI:** 10.5256/f1000research.17400.r39415

**Published:** 2018-10-24

**Authors:** Tony J. C. Harris

**Affiliations:** 1 Department of Cell & Systems Biology, University of Toronto, Toronto, ON, Canada

This paper describes the distribution of E-cadherin and the packing of cells within the developing epidermis of the zebrafish embryo. From 24-72 hours post fertilization the total amount of E-cadherin detected at all cell-cell contacts of the epidermis was found to increase. Over this same developmental period, hexagonal cells seemed to become more prevalent while tetragonal, octagonal and round cells become less frequent. Other parameters were quantified but were found not to be statistically significant between stages of development.

 A major concern with the analysis is the variability of the E-cadherin detection across the epidermis in single images (e.g. Figure 1e-h). The source of this variability was unclear. It was also unclear how the authors dealt with this variability for the quantifications. 

 The authors also noted “dot labeling in the cytoplasm” in panel 1d, but these puncta were not apparent in panel 1g which displayed the same developmental stage.

 Statistical tests were not applied to the analyses of polygon classes in Figure 3.  Also it was not stated whether SD or SE bars are shown in Figure 3b.

I have read this submission. I believe that I have an appropriate level of expertise to confirm that it is of an acceptable scientific standard, however I have significant reservations, as outlined above.

# Response

**Published:** 2018-10-25

**Authors:** Valeria Sigot

**Affiliations:** Universidad Nacional de Entre Ríos, Argentina

This paper describes the distribution of E-cadherin and the packing of cells within the developing epidermis of the zebrafish embryo. From 24-72 hours post fertilization the total amount of E-cadherin detected at all cell-cell contacts of the epidermis was found to increase. Over this same developmental period, hexagonal cells seemed to become more prevalent while tetragonal, octagonal and round cells become less frequent. Other parameters were quantified but were found not to be statistically significant between stages of development.

**A major concern with the analysis is the variability of the E-cadherin detection across the epidermis in single images (e.g. Figure 1e-h). The source of this variability was unclear.**

VS: If I understand well, your concern refers to the variability of E-cadh detection between stages and as well within single images (in fig 1 panel e-h). In this figure, single images (stages) are displayed as *maximum intensity projections* of 50 optical sections, in addition, they are larger than the ROIs used for quantitation aiming to show the curvature of the trunk and changes in cell morphology and density. As stated in the legend, they are contrast enhanced for visualization purposes. For quantitation, these and other images acquired were processed following the protocol (Suppl. File 1) and displayed in Figure 2, each panel representing a single ROI 3D of 50x50x6.6µm but here as the *“sum of intensities*” for 20 slices from which fluorescence intensity values were quantitated.

**It was also unclear how the authors dealt with this variability for the quantifications.**

VS: This was the main challenge of the approach because of the trunk morphology which changes during the studied period and the endogenous fluorescence of the yolk. Although the regions were selected at random along the trunk our criteria was to select the maximum ROI volume that cover a relatively flat region of the epidermis bilayer, with a minimum of 50 stacks including sections below and above the bilayer, to allow for proper restoration of fluorescence upon de-convolution and selecting regions from the yolk extension to the tail to minimize the contribution of the yolk fluorescence.

VS: The LMM (Linear Mixed Model) was employed to analyse the statistical differences of the quantitated intensity values between stages for the non-normal distribution of the data. This was selected as a suitable model to consider the variability of E-cadh detection between embryos within each stage. In this approach, the number of embryos per stage (5) and the number of ROIs/embryo (6) were set as a random effect and the stages (4) set as the fixed effect.

**The authors also noted “dot labeling in the cytoplasm” in panel 1d, but these puncta were not apparent in panel 1g which displayed the same developmental stage.**

VS: I have already replaced the image for a more evident one, to show the E-cadh cytoplasmic dot pattern along the trunk at 48 hpf.

**Statistical tests were not applied to the analyses of polygon classes in Figure 3.  Also it was not stated whether SD or SE bars are shown in Figure 3b.**

MFS: Figure 3 shows the distribution of all the polygon classes found in the EVL, in the studied stages. Although the pie chart displays the percentage of x-sided polygons, this represents a qualitative observation of the results and as you suggest, it would be more convenient, to support our results, to make a bar graph showing the mean values per n with its corresponding dispersion measure (SD; Standard Deviation). It is intended to show that there are significant differences between the main classes of polygons found, applying the corresponding statistical tests.

MFS: It will be necessary to modify the legend of this figure, specifying that Figure 3a corresponds to a bar graph displaying mean percentages of x-sided polygons of embryos at 24 hpf (n = 81 cells), 31 hpf (n = 147 cells), 48 hpf (n = 133 cells) and 72 hpf (n = 102 cells). The bars will represent mean values with their standard deviations. The type of statistical analysis performed on the data and the p-value obtained will be specified.

MFS: Regarding Figure 3b this shows the mean area of cell types at 24 hpf (EVL n = 73), 31 hpf (EVL n = 147), 48 hpf (EVL n = 133) and 72 hpf (EVL n = 102). In the same way, it will be necessary to complete this legend with statistical test applied, the obtained p-value and indicate that the bars represent cell mean area and its standard deviations (SD). All the data to build these figures were obtained from 30 3D-ROIs in five animals for each stage.

 The excel sheet that shows all these values (mean ± standard deviation) and the new Figure 3 will be completed and uploaded in "data availability" section.
